# Survey data on e-Procurement adoption in the Nigerian building industry

**DOI:** 10.1016/j.dib.2018.03.089

**Published:** 2018-03-28

**Authors:** Eziyi O. Ibem, Egidario B. Aduwo, Uwakonye O. Uwakonye, Patience F. Tunji-Olayeni, Emmanuel A. Ayo-Vaughan

**Affiliations:** a Department of Architecture, College of Science and Technology, Covenant University, KM 10 Idiroko, Road, Canaanland, Ota, Ogun State, Nigeria; b Department of Building Technology, College of Science and Technology, Covenant University, KM 10 Idiroko, Canaanland, Ota, Ogun State, Nigeria; c Department of Architecture, Bells University of Technology, Bells Drive, Ota, Ogun State, Nigeria

**Keywords:** e-Procurement, Building industry, Survey, Nigeria

## Abstract

This article is a description of data related to the research article entitled “Factors influencing e-Procurement adoption in the Nigerian Building Industry” (Ibem et al., 2016) [1]. The data were derived via a questionnaire survey involving 213 participants comprising architects, quantity surveyors, builders, construction/project managers, and engineers working in consulting and contracting firms, private client organisations and government agencies. The survey was conducted in Nigeria between June and November 2015. The data set contains responses on the levels of awareness and extent of use of the different e-Procurement technologies, the barriers to the uptake as well as the factors influencing e-Procurement adoption by organizations in the Nigerian building industry. The survey data and results of the analysis are made available in this article for further use by researchers; and for the purpose of improving understanding of the key findings of the survey.

**Specifications Table**

**Table:** 

Subject area	Construction Management
More specific subject area	e-Commerce adoption in construction
Type of data	Table
How data was acquired	Field Survey
Data format	Processed
Experimental factors	Both purposive and random sampling techniques were used in the selection of respondents in the survey
Experimental features	The data set was extracted from architects, builders, quantity surveyors, engineers, construction/project managers and procurement officers in consulting and contracting firms, client organisations and government agencies.
Data source location	Nigeria
Data accessibility	The data set is with this article

**Value of the data**

• The data provide empirical evidence on the current state of awareness and adoption of e-Procurement in the Nigerian building industry.
• From the data, researchers can gain fresh insight into the actual users of e-Procurement amongst the different professionals and organisations in the Nigerian building industry.
• The data set is valuable in improving understanding of the various factors that can have both positive and negative influence on the adoption of e-Procurement by originations in the Nigerian building industry.
• The data set also provides clues on the strategies to engage in ensuring a critical mass uptake of e-Procurement and maximising its benefits in the Nigerian building industry.
• The questionnaire instrument used in generating the data set can also be used for further research by other researchers who have interest on this subject.

## 1 Data

The data set contains responses obtained from a questionnaire survey of architects, builders, quantity surveyors, engineers, construction/project managers and procurement officers working in consulting and contracting firms, client organisations and government agencies who are directly involved in the procurement of building, materials, equipment/machineries, works and services in Nigeria. The questionnaire used was designed to elicit responses from the aforementioned stakeholders on a number of key issues related to e-Procurement use in the Nigerian building industry. Specifically, the data set shared in this article captures the responses of the participants on several issues, including (i) the respondents’ profiles and that of their organisations (ii) the respondents’ level of awareness of e-Procurement in construction (iii) the proportion of users to non-users of e-Procurement amongst the respondents in the survey (iv) the extent of use of the different e-Procurement technologies at the design, tendering and construction phases of building projects (v) barriers to the uptake of e-Procurement; and (vi) the technological, environmental and organisational factors that influenced the extent of adoption of e-Procurement in the Nigerian building industry. Analysis of the data (responses provided by participants in the questionnaire survey) can provide fresh insight into the aforementioned six key aspects covered in the survey of key stakeholders in the Nigerian building industry. Appendix A contains the sample of the questionnaire used. The SPSS file and results of the analyses of the responses are also shared in this article.

## 2 Experimental design, materials and methods

As stated earlier the data set shared in this article was sourced via a cross-sectional survey of key stakeholders in the Nigerian building industry. Previous studies [2], [3], [4], [5], [6] have adopted similar approach in obtaining empirical data from construction industry stakeholders. A structured and pre-tested questionnaire was used to elicit responses from architects, builders, quantity surveyors, engineers, construction/project managers and procurement officers in consulting and contracting firms, client organisations and government agencies who are directly involved in the procurement of building projects in Nigeria [1]. A sample of the questionnaire used is presented in Appendix A. A careful examination of the questionnaire reveals that it has three main sections. Section A was used to extract data on the professional roles of the respondents and their organizations, their levels of awareness of e-Procurement in construction, and the extent of use of the different categories of e-Procurement. Section B had questions on 29 different factors considered important in the decision to adopt e-Procurement by the respondents’ organizations. The data were extracted using 5-Likert type scale, where 1 is for *“Not Important”; 2* is for *“Least Important”;* 3 represents *“Undecided”;* 4 is for *“Important”;* and 5 represents *“Most’.* The last part of the questionnaire -Section C, was used to gather data on 26 factors that have adverse impact or are barriers to e-Procurement adoption in the organisations. Similarly, data on the barriers were derived using 5-Likert type scale, where *1= ”Has No Significant Effect”; 2 = “Has Very Little Effect”; 3= “Undecided” 4 = “ Has Significant Effect” 5 = “ Has the Most Significant Effect”.*

Before the main survey the questionnaire was pre-tested among 30 purposively selected respondents in architecture and quantity surveying firms, as well as client organizations in Lagos southwest Nigeria. Feedback from the pilot survey was used to fine-tune the final version of the questionnaire used in the main survey. In addition, the reliability of the scale of measurements used in Sections (B and C) of the questionnaire was investigated using Cronbach's alpha coefficient test. The tests returned Cronbach's alpha values of 0.851 and 0.834 for items in Section B and Section C, respectively. Since these values are more than 0.7 recommended in the research literature [7], the questionnaire instrument was considered reliable and consistent in measuring what it was designed for.

Part of the data set shared in this article was extracted from respondents in architectural firms in June 2015 during the 2015 Architects’ Colloquium in Abuja. The data from the builders were collected at Annual Builders’ National Conference and Meeting at the University of Ibadan in August 2015, while another part of the data set was generated from a survey of Quantity Surveying firms who participated at the Annual QS Research Conference at the Federal University of Technology, Akure in November 2015. In addition, client organizations, comprising government agencies, multinationals (e.g. oil and gas, telecommunication, manufacturing and building construction companies) in Lagos, Abuja and Port Harcourt were also purposively selected to participate in the research. In the administration of the questionnaire, the respondents were randomly selected; and to ensure that only one respondent from an organization was included in the survey, the respondents were asked to provide the names and locations of their organizations. Although, 500 questionnaires were distributed, 213 valid questionnaires representing around 43% of the total number of questionnaires distributed were retrieved and found useful. The data were analysed with the help of Statistical Package of the Social Sciences (SPSS) Version 20.

## Footnotes

Appendix A Supplementary data associated with this article can be found in the online version at 10.1016/j.dib.2018.03.089.
