# Supplementary material for: Survey data on e-Procurement adoption in the Nigerian building industry
Source: Data Brief. 2018 Mar 28;18:823–6. doi: 10.1016/j.dib.2018.03.089 (PMC5996743; doi:10.1016/j.dib.2018.03.089)
Supplement: Supplementary file 1 — Supplementary material [file mmc1.doc]

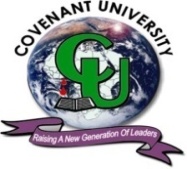


Eziyi O. Ibem; *Ph.D (Architecture); M.Sc (Arch); B.Sc (Arch) mnia, mnehr*

*Professor of Architecture*

Department of Architecture, Covenant University, Ota, Ogun State, Nigeria

[ibem.eziyi@covenantuniversity.edu.ng](mailto:ibem.eziyi@covenantuniversity.edu.ng) ; [eziyioffia@yahoo.com](mailto:eziyioffia@yahoo.com).

+234(0)8037779415; 08189892900

26^th^ January 2017

**Editor, Data in Brief**

**Conflict of Interest**

This is to declare that there is no conflict of interest among the authors of the manuscript entitled ‘*Survey Data on e-Procurement Adoption in the Nigerian Building Industry’* submitted for consideration in Data in Brief*.*

The authors have read the final draft and agreed that the manuscript be sent for review in this journal.

Thank you.


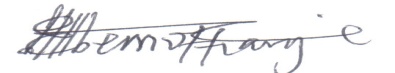


**Prof. Eziyi O. Ibem**; *Ph.D;*

*Corresponding Author*
