# Supplementary material for: Survey data on e-Procurement adoption in the Nigerian building industry
Source: Data Brief. 2018 Mar 28;18:823–6. doi: 10.1016/j.dib.2018.03.089 (PMC5996743; doi:10.1016/j.dib.2018.03.089)
Supplement: Supplementary file 3 — Supplementary material [file mmc3.docx]

**Appendix A**


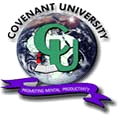


Covenant University, Km 10 Idiroko Road, Canaan Land, Ota, Ogun State Nigeria

**Questionnaire**

Dear Respondent,

The Built Environment Research Cluster of the School of Environmental Sciences, Covenant University, Ota, would like to invite you to take part in a research on the use of electronic procurement in the Nigerian construction industry. E-procurement refers to the use of electronic communications to buy services, goods and works or conduct tendering for construction works. The research seeks to ascertain the state of e-procurement use in the construction industry; and how the use of e-procurement technologies can be diffused and its benefits maximised in the Nigerian construction sector. All information provided will be treated with strict anonymity and used only for academic purposes.

Thank you for engaging with us in this research.

**Section A**

**Please tick (√) as appropriate**

1. Please indicate your role in the construction industry; Architect ( ) Builder ( ) Engineer ( ) Contractor ( ) Construction/ Project Manager ( ) Quantity Surveyor ( ) Procurement/ Supply Chain Manger ( ) Others, please specify---------------------

1. In which of the following categories of organizations are you employed? Consulting Firm ( ) Contractor ( )

Client organization ( ) Government Ministry/Parasatals/ Institution ( )

Others, please specify----------------------------------------------------------------------

1. Your construction procurement experiences are mostly in the Public Sector ( ) Private Sector ( ) Both Public and Private Sectors ( )?
2. What is the staff strength of your organization? Below 20 persons ( ) 20-50 Persons ( ) 51-100 persons ( ) More than 100 persons ( )
3. How many years have your organization been in business? Below 5years ( ) 6-10years ( )10 years + ( )
4. How many offices do you have in Nigeria? 1 ( ) 2 ( ) 3 ( ) More than 3 offices ( )
5. What is the range of your organization’s annual turnover? Less than N100 million ( ) N 100m- N 500 Million ( ) N 600m- N1billion ( ) Over N1billion ( )
6. Are you aware of the use of electronic procurement in construction? Yes ( ) No ( )
7. Which of these e-Procurement service providers does your organization subscribe to? Ariba ( ) Oracle ( ) Alibaba ( ); Others, Please specify-------------------------------------------------------------------------
8. How did you hear about e-Procurement in construction? Professionals Associations ( ) Mass Media ( ) Workshop/Conferences ( ) Vendors of e-Procurement technologies ( ) Business Associates ( )
9. How long have you been using e-Procurement in your organization? Less than 1 year ( ) 1-5years ( ) 6-10years ( ) More than 10years ( )
10. Have your oraganisation participated in construction project (s) that involved the use of e-procurement packages?

Yes ( ) No ( )

|  | **Please indicate how often you use these technologies and tools to carry out the following procurement activities. (** *Where* ***1****= Never used ;* ***2****= Used Sometimes; 3= Used Always)* | **1** | **2** | **3** |
| --- | --- | --- | --- | --- |
| 1 | **Obtain project briefs from Clients/ Give out project briefs to consultants** |  |  |  |
| a | E-mail Attachment |  |  |  |
| b | Internet-Supported Faxing |  |  |  |
| c | Voice of Internet Protocol ( e.g. Skype) |  |  |  |
| 2 | **Prepare construction drawings and documents** |  |  |  |
| a | Revit Architecture |  |  |  |
| b | ArchiCAD |  |  |  |
| c | Sketch up |  |  |  |
| d | AUTOCAD |  |  |  |
| e | VariCAD |  |  |  |
| f | Vectorworks |  |  |  |
| g | DraftSight |  |  |  |
| h | SolidWorks |  |  |  |
| i | CGS Revit Tools |  |  |  |
| j | RealCAD |  |  |  |
| k | Others, Please Specify |  |  |  |
| 3 | **Project Cost Estimation and Analysis** |  |  |  |
| a | CAD Quantity- Takeoff software packages |  |  |  |
| b | BIM-based Construction materials Quantity Takeoff Software |  |  |  |
| c | E-Catalogues |  |  |  |
| d | Others, please Specify |  |  |  |
| 3 | **Communication with Project Team Members** |  |  |  |
| a | Local Area Network (LAN) e.g. Intranet |  |  |  |
| b | Internet –based technologies ( e.g e-Mail, Face book, Slideshare) |  |  |  |
| c | Web-based integration and Collaboration Software Application Packages |  |  |  |
| c | Wireless Network |  |  |  |
| d | Others, Please Specify |  |  |  |
| 4 | **Project Meetings** |  |  |  |
| a | Teleconferencing |  |  |  |
| b | Video Conferencing |  |  |  |
| 5 | **Prepare of Tender Offers or Expression of Interest** |  |  |  |
| a | CAD Quantity Takeoff software Applications |  |  |  |
| b | BIM-based Construction materials Quantity Takeoff Software |  |  |  |
| c | E-Catalogues (E-Market) |  |  |  |
| 6 | **Submit of Tender offers or Expression of interest** |  |  |  |
| a | Cloud-based Systems and Applications ( e.g. Microsoft SharePoint) |  |  |  |
| b | Project Portals |  |  |  |
| c | E-mail Attachment |  |  |  |
| d | Internet-based systems /Software applications |  |  |  |
| e | CDROM |  |  |  |
| f | Others, Please specify-- |  |  |  |
| 7 | **Work Scheduling** |  |  |  |
| a | Web-Enabled Project Management Software Applications |  |  |  |
| b | BIM Technology |  |  |  |
| c | Microsoft Project |  |  |  |
| d | Others please specify........... |  |  |  |
| 8 | **Search for, and Select Materials and Equipment from Suppliers** |  |  |  |
| a | E-Market Place |  |  |  |
| b | Internet-based Geographic Information System (GIS) |  |  |  |
| c | Company Website |  |  |  |
| d | e-Catalogues |  |  |  |
| 9 | **Purchase and/or Hire of Construction Equipment and Materials** |  |  |  |
| a | Electronic Data Exchange (EDI) |  |  |  |
| b | E-Market Places |  |  |  |
| c | Electronic Reverse auctioning |  |  |  |
| d | Video Conferencing |  |  |  |
| 10 | **Track the movement of materials from Suppliers to construction sites** |  |  |  |
| a | Wi-Fi Networks (e.g. cellular modems; phones) |  |  |  |
| b | Radio Frequency Identification (RFID) |  |  |  |
| c | Barcode Technology |  |  |  |
| 11 | **Handle of Materials on Project Sites** |  |  |  |
| a | Geographic Positioning System (GPS) |  |  |  |
| 12 | **Monitor the Progress of Work on project sites** |  |  |  |
| a | BIM Technology |  |  |  |
| b | Web-Enabled Project Management Software Applications |  |  |  |
| c | Microsoft Project |  |  |  |
| d | Web-based Project Camera |  |  |  |
| e | 3D Scanner or LADAR ( Laser Distance and Ranging) Technology |  |  |  |
| f | Internet-enabled Multimedia Technology |  |  |  |
| 13 | **Track Project Cost and Scheduling** |  |  |  |
| b | Radio Frequency Identification (RFID) |  |  |  |
| c | BIM Technology |  |  |  |
| 14 | **Make/ Receive of Payment for construction goods , works and services** |  |  |  |
| a | Credit Card |  |  |  |
| b | Electronic fund Transfer (EFT) |  |  |  |
| c | Others, Please Specify |  |  |  |

**B: Factors influencing the use of e-Procurement in Construction**

| **Please rate the following in the order of their importance on the decision of your organization to use e-Procurement in construction s [***1= “Not Important”; 2 = “Least Important”; 3= “Undecided” 4 = “Important” 5 = “Most Important”]* | | | | | | |
| --- | --- | --- | --- | --- | --- | --- |
|  |  | **1** | **2** | **3** | **4** | **5** |
| 1 | Availability of e-Procurement Packages |  |  |  |  |  |
| 2 | The cost of acquiring and operating the packages |  |  |  |  |  |
| 3 | The extent to which e-Procurement technologies and tools are easy to use |  |  |  |  |  |
| 4 | Availability of IT staff/manpower in my organization |  |  |  |  |  |
| 5 | The compatibility of e-Procurement with our existing procurement processes |  |  |  |  |  |
| 6 | Result of the initial attempt to use e-Procurement in my organization |  |  |  |  |  |
| 7 | The number of existing users amongst my business partners |  |  |  |  |  |
| 8 | The decision by our clients/service providers to use e-Procurement |  |  |  |  |  |
| 9 | The desire to align with the global trend in e-Procurement use |  |  |  |  |  |
| 10 | What early users said about e-Procurement technologies and tools |  |  |  |  |  |
| 11 | The geographical spread of the business activities of my organization |  |  |  |  |  |
| 12 | Scope of the operational activities of my organization |  |  |  |  |  |
| 13 | Level of business activities in the construction industry in the country |  |  |  |  |  |
| 14 | Financial base of my organization |  |  |  |  |  |
| 15 | Sizes of my organization |  |  |  |  |  |
| 16 | Top management support resulting from perceived overall benefits procurement use |  |  |  |  |  |
| 17 | The benefits of reduction in time spent on procurement process |  |  |  |  |  |
| 18 | The benefits of competitiveness inherent in e-Procurement |  |  |  |  |  |
| 19 | The benefits of enhanced level of efficiency in job delivery |  |  |  |  |  |
| 20 | The involvement of less paper work in e-Procurement |  |  |  |  |  |
| 21 | Less labour intensive feature of e-Procurement |  |  |  |  |  |
| 22 | The benefits of reduction in errors associated with paper-based methods |  |  |  |  |  |
| 23 | The benefits of good inventory management/ record keeping |  |  |  |  |  |
| 24 | The increase in profit margin associated with e-procurement |  |  |  |  |  |
| 25 | The benefits of effective communication between project team members |  |  |  |  |  |
| 26 | The benefits of easier coordination of procurement activities |  |  |  |  |  |
| 27 | The potential of e-procurement to improves efficiency of collaboration among project team |  |  |  |  |  |
| 28 | The benefits of visibility of our business inherent in e-Procurement use |  |  |  |  |  |
| 29 | The benefits of elimination of geographic barrier in procurement process |  |  |  |  |  |

**C: Factors Adversely Affecting the Maximization of the Benefits of e-Procurement in Construction**

| **Please rate the following factors in order of their adverse impact on e-Procurement use in construction [** ***1****= “Has No Significant Effect”;* ***2*** *= “Has Very Little Effect”;* ***3****= “Undecided”* ***4*** *= “ Has Significant Effect”* ***5*** *= “ Has The Most Significant Effect”]* | | | | | | |
| --- | --- | --- | --- | --- | --- | --- |
|  |  | **1** | **2** | **3** | **4** | **5** |
| 1 | The benefits of using e-Procurement in construction are not very clear |  |  |  |  |  |
| 2 | Technical challenges associated with the transition from paper-based method to e-Procurement |  |  |  |  |  |
| 3 | Lack of widely accepted e-Procurement software solution in construction |  |  |  |  |  |
| 4 | Lack of technical expertise to handle e-Procurement technologies |  |  |  |  |  |
| 5 | The complicated nature and process involved in e-Procurement use |  |  |  |  |  |
| 6 | Unreliable power supply situation in Nigeria |  |  |  |  |  |
| 7 | Poor Internet and ICT Infrastructure in Nigeria |  |  |  |  |  |
| 8 | High cost of investment in e-Procurement Technologies and Tools |  |  |  |  |  |
| 9 | Lack of interoperability of e-Procurement software packages |  |  |  |  |  |
| 10 | Lack of uniform standards in the use of e-Procurement packages |  |  |  |  |  |
| 11 | Safety and Security issues in e-Procurement transactions |  |  |  |  |  |
| 12 | Lack of confidentially in e-Procurement transactions |  |  |  |  |  |
| 13 | Delays in the transmission of data and information |  |  |  |  |  |
| 14 | Concerns over the legality of electronic contracts |  |  |  |  |  |
| 15 | Lack of a National policy on e-Procurement in Nigeria |  |  |  |  |  |
| 16 | Lack of forum to exchange ideas on the use of e-Procurement |  |  |  |  |  |
| 17 | Inadequate government support for e-Procurement in construction |  |  |  |  |  |
| 18 | Lack of universal format and standard in which construction materials are described, displayed and specified |  |  |  |  |  |
| 19 | General resistance to change by people in the construction industry |  |  |  |  |  |
| 20 | Lack of flexibility in the use of e-Procurement |  |  |  |  |  |
| 21 | Relatively low human-to-human contact in e-Procurement transactions |  |  |  |  |  |
| 22 | Lack of awareness on e-Procurement in the country |  |  |  |  |  |
| 23 | The fear for loss of jobs and staff turnover |  |  |  |  |  |
| 24 | Lack of top management support |  |  |  |  |  |
| 25 | Inaccurate display of data and information at the receiver’s end |  |  |  |  |  |
| 26 | The fear that e-Procurement will help curb corruption in the industry |  |  |  |  |  |

Please, kindly suggest how the benefits of e-Procurement in construction can be maximised in the Nigerian construction industry.

**--------------------------------------------------------------------------------------------------------------------------------------------**

..........................................................................................................................................................................................

...........................................................................................................................................................................................

If you would like to engage with us further in research, please provide us with your contact details below

Name of your Organization:--------------------------------------------------------------------------------------------------------

Location:--------------------------------------------------------------------------------------------------------------------------------

E-Mail Address:-------------------------------------------------------------------------------------------------------------------------
