# Supplementary material for: Survey data on e-Procurement adoption in the Nigerian building industry
Source: Data Brief. 2018 Mar 28;18:823–6. doi: 10.1016/j.dib.2018.03.089 (PMC5996743; doi:10.1016/j.dib.2018.03.089)
Supplement: Supplementary file 2 — Supplementary material [file mmc2.zip › Tables.doc]

**Table 1**

Profile of the Respondents and their Organizations

| Attributes | N=213 | Percentage |
| --- | --- | --- |
| **Role/ Duty/ profession** |  |  |
| Architect | 75 | 35.2 |
| Builder | 47 | 22.1 |
| Engineer | 6 | 2.8 |
| Contractor | 3 | 1.4 |
| Construction/ Project Manager | 20 | 9.4 |
| Quantity Surveyor | 56 | 26.3 |
| Procurement/Supply Chain manager | 1 | 0.5 |
| Others | 5 | 1.4 |
| **Type of Organization** |  |  |
| Consulting firms | 72 | 33.8 |
| Contractors | 45 | 21.1 |
| Client organization | 11 | 5.2 |
| Government Ministry/ Parasatals | 75 | 35.2 |
| Academic Institutions | 10 | 4.7 |
| **Sector of Procurement Experience** |  |  |
| Public sector only | 36 | 16.9 |
| Private Sector only | 52 | 24.4 |
| Both Public and private | 125 | 58.7 |
| **Staff Strength ( Persons)** |  |  |
| Below 20 | 94 | 44.1 |
| 20-50 | 45 | 21.1 |
| 51-100 | 14 | 6.6 |
| More than 100 | 56 | 26.3 |
| No Response | 4 | 1.9 |
| **Age of Organization** |  |  |
| Below 5 years | 31 | 14.6 |
| 6-10 years | 52 | 24.4 |
| More than 10 years | 126 | 59.2 |
| No Response | 4 | 1.9 |
| **Number of Offices in Nigeria** |  |  |
| One | 84 | 39.4 |
| Two | 52 | 24.4 |
| Three | 17 | 8.0 |
| More than three | 42 | 19.7 |
| No Response | 18 | 8.5 |
| **Range of annual turnover in Naira** |  |  |
| Less than 100 million | 91 | 42.7 |
| 100-500 million | 40 | 18.8 |
| 600 million-1bilion | 17 | 8.0 |
| Over 1 billion | 42 | 19.7 |
| No Response | 23 | 10.8 |

Table 2

Software Packages used in Design and Cost Estimation of Building Projects

| **Procurement Task** | **Software Package** | **Frequency of Use** | **n =213** | **Percentage** |
| --- | --- | --- | --- | --- |
| **Design of Project** | *Revit* | Never used | 101 | 47.5 |
|  |  | Sometimes | 53 | 24.9 |
|  |  | Always | 59 | 27.7 |
|  | *ArchiCAD* | Never used | 100 | 47.0 |
|  |  | Sometimes | 53 | 24.9 |
|  |  | Always | 60 | 28.2 |
|  | *Sketch Up* | Never used | 105 | 54.0 |
|  |  | Sometimes | 54 | 25.4 |
|  |  | Always | 44 | 20.7 |
|  | *AUTOCAD* | Never used | 30 | 14.1 |
|  |  | Sometimes | 40 | 18.8 |
|  |  | Always | 143 | 67.1 |
|  | *VariCAD* | Never used | 175 | 82.2 |
|  |  | Sometimes | 25 | 11.7 |
|  |  | Always | 13 | 6.1 |
|  | *DraftSight* | Never used | 178 | 83.6 |
|  |  | Sometimes | 26 | 12.2 |
|  |  | Always | 9 | 4.2 |
|  | *Vectorworks* | Never used | 179 | 84.1 |
|  |  | Sometimes | 26 | 12.2 |
|  |  | Always | 8 | 3.8 |
|  | *Solid Works* | Never used | 180 | 85.0 |
|  |  | Sometimes | 24 | 11.3 |
|  |  | Always | 9 | 4.2 |
|  | *CGS Revit Tools* | Never used | 171 | 80.3 |
|  |  | Sometimes | 31 | 14.6 |
|  |  | Always | 11 | 5.2 |
|  | *RealCADD* | Never used | 183 | 86.0 |
|  |  | Sometimes | 25 | 11.7 |
|  |  | Always | 5 | 2.3 |
| **Project Cost Estimation** | *BIM-Based construction materials , Quantity takeoff software* | Never Used | 141 | 66.2 |
|  |  | Sometimes | 55 | 25.8 |
|  |  | Always | 17 | 8.0 |
|  | *CAD-based Quantities, Take-off software applications* | Never used | 121 | 56.8 |
|  |  | Sometimes | 53 | 24.9 |
|  |  | Always | 39 | 18.3 |
|  | *Other quantity & cost estimation software (e.g.Masterbill (MB3+) , QS Elite or Win QS* | Never used | 130 | 61.0 |
|  |  | Sometimes | 13 | 6.1 |
|  |  | Always | 70 | 32.9 |
|  | *E - Catalogues* | Never used | 85 | 39.9 |
|  |  | Sometimes | 110 | 51.7 |
|  |  | Always | 18 | 8.5 |

Table 3

e- Tendering Tools and Applications Used by the Respondents

| Procurement Task | Technologies | Frequency of Use | n =213 | % |
| --- | --- | --- | --- | --- |
| **Exchange of project briefs and specifications** | *E-mail Technology* | Never used | 45 | 21.1 |
|  |  | Sometimes | 74 | 34.7 |
|  |  | Always | 94 | 44.1 |
|  | *Clients website* | Never used | 78 | 36.6 |
|  |  | Sometimes | 72 | 33.8 |
|  |  | Always | 63 | 29.6 |
| **Submission of Expression of Interest, Proposals or Tender offers** | *E-mail Technology* | Never used | 64 | 30.0 |
|  |  | Sometimes | 56 | 26.3 |
|  |  | Always | 93 | 43.7 |
|  | *Project Portal* | Never used | 137 | 64.3 |
|  |  | Sometimes | 58 | 27.2 |
|  |  | Always | 18 | 8.5 |
|  | *Cloud-based systems and applications (e.g Microsoft SharePoint, Dropbox)* | Never used | 155 | 72.8 |
|  |  | Sometimes | 40 | 18.8 |
|  |  | Always | 18 | 8.5 |
|  | *Internet-based systems/software applications* | Never used | 102 | 48.9 |
|  |  | Sometimes | 61 | 28.6 |
|  |  | Always | 50 | 23.5 |
|  | *Compact (CD)/ digital video disc (DVD)* | Never used | 101 | 52.1 |
|  |  | Sometimes | 59 | 27.7 |
|  |  | Always | 43 | 20.2 |
|  | *Electronic Data Interchange (EDI)* | Never used | 148 | 70.5 |
|  |  | Sometimes | 45 | 21.1 |
|  |  | Always | 20 | 9.4 |
| **Evaluation and Selection of expression of interests, proposals and tender offers** | *Electronic reverse auctioning* | Never used | 213 | 100 |
|  |  | Sometimes | 0 | 0.0 |
|  |  | Always | 0 | 0.0 |
|  | *Voice over Internet Protocol (VOIP)* | Never used | 169 | 79.3 |
|  |  | Sometimes | 30 | 14.1 |
|  |  | Always | 14 | 6.6 |
|  | *Internet-based Tendering software (e.g. DecisionMax Software).* | Never used | 163 | 76.5 |
|  |  | Sometimes | 15 | 7.0 |
|  |  | Always | 35 | 16.4 |

Table 4

e-Procurement Technologies used at Construction Stage of Building Projects

| Procurement Task | Application | Frequency of Use | n =213 | % |
| --- | --- | --- | --- | --- |
| **Communication with Project Team Members** | *E-mail technology* | Never used | 5 | 2.4 |
|  |  | Sometimes | 71 | 33.3 |
|  |  | Always | 137 | 64.3 |
|  | *Web 2.0 technology ( e.g) Social Media Platforms* | Never used | 50 | 23.5 |
|  |  | Sometimes | 55 | 25.8 |
|  |  | Always | 108 | 50.7 |
|  | *Voice over Internet Protocol (VOIP)* | Never used | 114 | 53.5 |
|  |  | Sometimes | 68 | 31.9 |
|  |  | Always | 31 | 14.6 |
|  | *Web-based Communication Apps* | Never used | 43 | 20.2 |
|  |  | Sometimes | 50 | 23.5 |
|  |  | Always | 120 | 56.3 |
| **Work Scheduling** | *Project management software application* | Never used | 140 | 65.8 |
|  |  | Sometimes | 42 | 19.7 |
|  |  | Always | 31 | 14.6 |
|  | *Microsoft Project* | Never used | 86 | 40.4 |
|  |  | Sometimes | 44 | 20.7 |
|  |  | Always | 83 | 39.0 |
|  | *Building Information Modelling (BIM)* | Never used | 131 | 61.5 |
|  |  | Sometimes | 60 | 28.2 |
|  |  | Always | 22 | 10.3 |
| **Searching for Building Materials and Equipment** | *E- Market Place* | Never used | 118 | 55.4 |
|  |  | Sometimes | 59 | 27.7 |
|  |  | Always | 36 | 16.9 |
|  | *Geographic information system (GIS)* | Never used | 118 | 55.4 |
|  |  | Sometimes | 57 | 26.8 |
|  |  | Always | 38 | 17.8 |
|  | *Company/ suppliers website* | Never used | 40 | 18.8 |
|  |  | Sometimes | 63 | 29.6 |
|  |  | Always | 110 | 51.6 |
| **Purchasing and Hiring of Building Materials Equipments** | *E-Market place* | Never used | 137 | 54.3 |
|  |  | Sometimes | 51 | 23.9 |
|  |  | Always | 25 | 11.7 |
|  | *Voice over Internet Protocol (VOIP)* | Never used | 169 | 79.3 |
|  |  | Sometimes | 30 | 14.1 |
|  |  | Always | 14 | 6.6 |
|  | *Web-based Project Management Systems (WPMS)* | Never used | 180 | 84.5 |
|  |  | Sometimes | 8 | 3.80 |
|  |  | Always | 25 | 11.74 |
|  |  | Always | 123 | 57.8 |
| **Tracking of the movement of materials from Suppliers to sites** | *Wireless networks e.g. cellular modems, phones* | Never used | 38 | 17.84 |
|  |  | Sometimes | 70 | 32.86 |
|  |  | Always | 105 | 49.30 |
|  | *Radio frequency identification (RFID)* | Never used | 173 | 81.2 |
|  |  | Sometimes | 32 | 15.0 |
|  |  | Always | 8 | 3.8 |
|  | *Barcode Technology* | Never used | 181 | 84.9 |
|  |  | Sometimes | 27 | 12.7 |
|  |  | Always | 5 | 2.4 |
|  | *Geographic positioning systems (GPS)* | Never used | 151 | 71.0 |
|  |  | Sometimes | 51 | 23.9 |
|  |  | Always | 11 | 5.2 |
| **Monitoring the Progress of work on construction Sites** | *Cloud-based BIM* | Never used | 149 | 70 |
|  |  | Sometimes | 43 | 20.2 |
|  |  | Always | 21 | 9.9 |
|  | *Web-enabled project management system* | Never used | 139 | 65.3 |
|  |  | Sometimes | 50 | 23.5 |
|  |  | Always | 24 | 11.3 |
|  | *Web-enabled digital cameras* | Never used | 147 | 69.0 |
|  |  | Sometimes | 47 | 22.1 |
|  |  | Always | 19 | 8.9 |
|  | *3D scanner or LADAR Technology* | Never used | 165 | 77.4 |
|  |  | Sometimes | 38 | 17.8 |
|  |  | Always | 10 | 4.7 |
|  | *Web- enabled multimedia technology* | Never used | 146 | 67.6 |
|  |  | Sometimes | 48 | 22.5 |
|  |  | Always | 19 | 8.9 |

.

Table 5

Descriptive statistics of Perceived Barriers to the Uptake of e-Procurement in the Nigerian Building Industry

| Barriers | Mean | Std. Deviation |
| --- | --- | --- |
| High cost of investment in e-Procurement technologies and tools | 4.44 | 4.90 |
| Lack of technical expertise to handle e-Procurement technologies | 4.12 | 3.18 |
| Poor Internet and ICT infrastructure in Nigeria | 4.05 | 1.04 |
| Unrealizable power supply situation in Nigeria | 4.06 | 1.13 |
| Safety and security issues in e-procurement transaction | 3.94 | 3.27 |
| Lack of uniform standard in the use of e-Procurement packages | 3.89 | 3.21 |
| Lack of interoperability of e-Procurement software packages | 3.86 | 3.27 |
| Inadequate government support for e-Procurement in construction | 3.81 | 1.09 |
| Lack of awareness on e-Procurement in the industry | 3.77 | 1.22 |
| Technical challenges associated with the transition paper based method to e-Procurement | 3.76 | 3.31 |
| Lack of a National policy on e-Procurement in Nigeria | 3.76 | 1.10 |
| Lack of forum to exchange ideas on the use of e-Procurement | 3.75 | 1.04 |
| Lack of top management support | 3.73 | 1.07 |
| General resistance to change by people in the construction industry | 3.63 | 1.16 |
| Lack of widely accepted e-Procurement software solutions in construction | 3.60 | 1.22 |
| The fear that e-Procurement will help curb corruption in the industry | 3.60 | 1.29 |
| The complicated nature and process involved in e-procurement use | 3.59 | 1.06 |
| Lack of universal format and standard in which construction materials are described, displayed and specified | 3.57 | 1.09 |
| Lack of confidentiality in e-Procurement transactions | 3.56 | 1.16 |
| The fear for loss of jobs and staff turnover | 3.54 | 1.19 |
| Concerns over the legality of electronic contracts | 3.48 | 1.10 |
| Inaccurate display of data and information at the receivers' end | 3.44 | 1.14 |
| Delays in the transmission of data and information | 3.42 | 2.53 |
| Lack of flexibility in the use of e-Procurement | 3.38 | 1.16 |
| The benefits of using e-Procurement in construction are not very clear | 3.30 | 1.28 |
| Relatively low human to human contact in e-Procurement transactions | 3.26 | 1.22 |

Table 6

Descriptive Statistics of Factors Influencing e-Procurement use in the Nigerian Building Industry

| Factors | Mean | Standard Deviation |
| --- | --- | --- |
| Benefits of enhanced level of efficiency in job delivery | 4.73 | 5.94 |
| Benefits of elimination of geographic barriers in procurement | 4.59 | 4.87 |
| Benefits of effective communication between project team members | 4.20 | 0.81 |
| Availability of IT manpower in my organization | 4.14 | 0.89 |
| Benefits of good inventory management and record keeping | 4.13 | 0.80 |
| The need to align with global trends in e-Procurement use in other industries | 4.13 | 1.10 |
| Reduction in errors associated with paper-based methods | 4.08 | 0.82 |
| Easier coordination of procurement activities | 4.04 | 0.92 |
| Reduction in time spent on procurement process | 3.99 | 1.06 |
| Less paper work | 3.99 | 0.99 |
| Cost of acquiring and operating the package | 3.97 | 1.15 |
| Efficiency of collaboration amongst project team | 3.97 | 0.92 |
| Availability of e-Procurement packages | 3.93 | 1.07 |
| Less labour intensive feature of e-Procurement | 3.90 | 0.92 |
| Decision by our clients/service providers to e-Procurement | 3.90 | 0.99 |
| Perceived benefits associated with reduction in the procurement cost | 3.89 | 1.03 |
| Compatibility of e-Procurement with our existing procurement process | 3.85 | 0.95 |
| Benefit of competitiveness inherent in e-Procurement | 3.82 | 0.95 |
| Increase in profit margin associated with e- Procurement | 3.80 | 1.00 |
| Visibility of our business inherent in e-Procurement | 3.77 | 1.04 |
| Financial base of my organization | 3.73 | 1.21 |
| Size of my organization | 3.71 | 1.14 |
| The extent to which e-procurement technology and tools are easy to use | 3.69 | 1.10 |
| Level of business activities in the construction industry in the country | 3.48 | 1.06 |
| Number of existing users amongst business partners | 3.45 | 1.14 |
| Geographical spread of the business activities of my organization | 3.42 | 1.15 |
| What early users said about e-Procurement technologies and tools | 3.42 | 1.24 |
| Result of the initial attempt at e-Procurement use in my organization | 3.25 | 1.26 |
