# Supplementary material for: Survey data on e-Procurement adoption in the Nigerian building industry
Source: Data Brief. 2018 Mar 28;18:823–6. doi: 10.1016/j.dib.2018.03.089 (PMC5996743; doi:10.1016/j.dib.2018.03.089)
Supplement: Supplementary file 2 — Supplementary material [file mmc2.zip › Figures.doc]

Figures

Figure 1: Level of Awareness of e-Procurement

Figure 2: Distribution of User of e-Procurement among Professional Consultants
